# Knowledge, Attitudes, and Practices of Primary Care Physicians Regarding Vaginal Discharge Management in Alahsa, Saudi Arabia

**DOI:** 10.7759/cureus.91275

**Published:** 2025-08-30

**Authors:** Zainab T Alramadhan, Albanderi A Albandar, Mousa M Aljassim

**Affiliations:** 1 Family Medicine, Alahsa Health Cluster, Alahsa, SAU; 2 Family Medicine, Al Mansorah Primary Care Center, Alahsa, SAU

**Keywords:** knowledge attitude practice, primary health care, saudi arabia, vaginal discharge, vulvovaginitis

## Abstract

Background: Vaginal discharge is one of the most common gynecological complaints among women of reproductive age. Its effective management depends on the knowledge, attitudes, and practices (KAP) of primary health care physicians. In regions such as Alahsa, Saudi Arabia, assessing physician competence is essential to improve patient outcomes and address cultural and systemic barriers to care.

Methods: A cross-sectional study was conducted in 2024 among 233 primary health care physicians in Alahsa. Participants were selected using a convenience sampling technique. Data were collected through a structured self-administered questionnaire covering demographics, knowledge of vaginal discharge etiologies and treatments, attitudes toward clinical management, and diagnostic and treatment behaviors. Data analysis included descriptive statistics, chi-square tests, and multivariate regression using SPSS.

Results: A total of 210 (90.1%) of participants demonstrated good knowledge, particularly regarding common etiologies and first-line treatments for bacterial vaginosis, vulvovaginal candidiasis, and trichomoniasis. However, diagnostic knowledge was more limited; only 96 (41.2%) correctly identified saline wet mount as a diagnostic tool for *Trichomonas vaginalis*. Attitudes were generally favorable, with strong support for guideline adherence and patient education. Marital status and professional level were significantly associated with knowledge levels (p < 0.05), while other sociodemographic factors were not. Clinical practices varied considerably, with limited use of physical examinations and diagnostic testing, often attributed to resource constraints or cultural sensitivities.

Conclusion: Primary health care physicians in Alahsa exhibit good knowledge and positive attitudes regarding the management of vaginal discharge. However, inconsistencies in clinical practice, particularly in diagnostic approaches, highlight the need for targeted training and improved diagnostic accessibility to enhance the quality of care.

## Introduction

Abnormal vaginal discharge is one of the most prevalent gynecological complaints among women of reproductive age presenting to primary care physicians [1-3]. Many women seek medical attention when these symptoms cause concern or negatively impact their quality of life [4,5].

Vaginal discharge can be classified as either physiological or pathological. The normal vaginal pH typically ranges from 3.8 to 4.4, and daily discharge volume may vary between individuals, estimated at 1-4 mL per day [6,7]. Healthy discharge is usually thick, clear, mucoid, and either odorless or mildly malodorous [8]. Its characteristics fluctuate during the menstrual cycle, especially during the ovulatory and luteal phases, under the influence of estrogen levels. Factors such as menstruation, puberty, and hormone replacement therapy also affect hormonal balance and, consequently, discharge characteristics [8]. Given that some variations are physiological while others are pathological, it is important for physicians to ask whether a patient has noticed changes in her usual vaginal discharge [3].

Pathological discharge typically presents with changes in color, consistency, odor, or volume, and is often associated with symptoms such as dysuria, pelvic discomfort, or pruritus. This constellation of symptoms is collectively referred to as vulvovaginitis [8]. Common risk factors for vulvovaginitis include antibiotic use, low estrogen levels, foreign bodies, hormonal contraceptives, poor hygiene practices, pregnancy, and other hormonal fluctuations [8]. Accurate identification of the underlying cause is essential for effective treatment. Etiologies can be broadly divided into infectious and non-infectious. Among infectious causes, bacterial vaginosis, vulvovaginal candidiasis, and trichomoniasis account for approximately 70% of cases [9].

Bacterial vaginosis alone accounts for up to 50% of vaginitis cases among women aged 14-49 years, irrespective of race or ethnicity. It has been associated with increased risk of HIV acquisition, other sexually transmitted infections (STIs), and adverse pregnancy outcomes such as miscarriage, preterm birth, and postpartum complications [10]. Non-infectious causes of vaginal discharge include genital malignancies (e.g., tumors of the vulva, vagina, cervix, fallopian tubes, or endometrium), atrophic vaginitis, contact dermatitis, and foreign body reactions [8].

Effective diagnosis requires a comprehensive clinical evaluation, including detailed history-taking, physical and speculum examination, and appropriate laboratory investigations. History should cover gynecologic, sexual, medical, and surgical backgrounds, as well as discharge characteristics and associated symptoms. Foreign bodies must be considered and ruled out. Unless contraindicated, all women presenting with abnormal discharge should undergo a speculum examination [8].

Due to limited in-house diagnostic facilities in many primary care centers, specimens are typically referred to external laboratories. Point-of-care tests, including pH-based methods using narrow-range paper (pH 4-7), can assist in identifying lower genital tract infections. A high vaginal swab for microscopy (wet mount and Gram stain), culture, and sensitivity testing is recommended for patients with recurrent or persistent symptoms [8].

Given the socio-cultural context and healthcare infrastructure in Alahsa, Saudi Arabia, additional challenges may influence the management of vaginal discharge. These include limited access to diagnostic resources, cultural sensitivities regarding reproductive health, and inconsistent adherence to clinical guidelines. This study aims to assess the knowledge, attitudes, and practices (KAP) of primary health care physicians in Alahsa in managing vaginal discharge and to identify key barriers to optimal care. The findings will help inform strategies to improve diagnostic accuracy, guideline adherence, and overall patient outcomes.

## Materials and methods

Study design, setting, and population

This study employed a cross-sectional design, enabling the collection of data at a single point in time to provide a snapshot of current KAP among primary care physicians. The study was conducted in primary health care centers located in Alahsa’a, a region in the Eastern Province of Saudi Arabia. The target population included all primary health care physicians practicing in Alahsa’a (N = 499), who are responsible for the initial diagnosis and management of patients presenting with vaginal discharge.

Sampling method

A non-probability convenience sampling technique was used. Based on the total population size, an initial sample size of 217 was estimated to achieve adequate statistical power. To account for potential non-response and enhance reliability, the sample size was increased to 230.

Data collection

Data were collected using a structured, self-administered questionnaire, developed by the authors based on the study objectives and relevant literature. The tool included sections on demographic characteristics, knowledge of vaginal discharge etiologies and treatments, attitudes toward management practices, and diagnostic and treatment behaviors. The full questionnaire is provided in the Appendix. It was distributed either electronically or in person, depending on physician accessibility, and follow-up reminders were sent to maximize the response rate.

Survey instrument

The questionnaire was adapted from previously validated tools and customized to align with the study objectives. It comprised sections on demographic characteristics, knowledge of vaginal discharge causes and treatments, attitudes toward management practices, and self-reported clinical behaviors. Knowledge items assessed recognition of clinical features and treatment options; attitudes were measured using a Likert scale; and practice questions covered diagnostic and treatment protocols.

Statistical analysis

Data analysis was performed using Statistical Package for the Social Sciences (IBM Corp., Armonk, NY). Descriptive statistics (frequencies, percentages, means, and standard deviations) summarized demographic variables and KAP responses. Inferential analyses, including chi-square tests and t-tests, were applied to identify associations between sociodemographic factors and KAP variables. Multivariate logistic regression was used to identify independent predictors of effective clinical practices.

Ethical considerations

Ethical approval for this study was granted by the Institutional Review Board (IRB) of the Maternity and Children’s Hospital - Al Ahsa (IRB Log No: 541224-EP-2024; IRB Registration No: H-05-HS-137) on December 2, 2024. The study received expedited review and clearance to proceed according to the approved protocol. Written informed consent was obtained from all participants prior to data collection. Confidentiality was strictly maintained by anonymizing responses, omitting any personal identifiers, and reporting findings in aggregate form. Participation was voluntary, and all respondents were informed of their right to withdraw at any stage without penalty.

## Results

The study included 233 participants with a mean age of 31.46 ± 6.22 years (range: 24-55 years). The sample was nearly gender-balanced, with 118 (50.6%) males and 115 (49.4%) females. Most participants were aged 20-29 years (n=114; 48.9%), followed by those aged 30-39 years (n=93; 39.9%). A majority were married (n=174; 74.7%), while 49 (21.0%) were single and 10 (4.3%) were divorced.

Regarding professional level, residents comprised the largest group (n=115; 49.4%), followed by specialists (n=45; 19.3%), general practitioners (n=42; 18.0%), and consultants (n=31; 13.3%). Most participants reported a “good” income (n=174; 75.1%), while (n=52; 22.3%) had a high income, and (n=6; 2.6%) reported a low income. The majority had 1-5 years of professional experience (n=151; 64.8%). Nearly all participants were Saudi nationals (n=228; 97.9%).

Table 1 presents the detailed sociodemographic characteristics of the study population.

**Table 1:** Sociodemographic Characteristics Age is presented in years. Income categories were defined as: low (<5,000 SAR/month), good (5,000–10,000 SAR/month), high (>10,000 SAR/month). GP = general practitioner; SD = standard deviation; PHC = primary health care ^*^Totals do not sum to 233 due to one participant not reporting income.

Variable	Sociodemographic Characteristics	Frequency	Percent
Gender	Male	118	50.6
	Female	115	49.4
Age (years)	20-29	114	48.9
	30-39	93	39.9
	40-49	22	9.4
	50-59	4	1.7
Marital status	Single	49	21
	Married	174	74.7
	Divorced	10	4.3
Professional level	GP	42	18
	Resident	115	49.4
	Specialist	45	19.3
	Consultant	31	13.3
Income^*^	Low	6	2.6
	Good	174	75.1
	High	52	22.3

Overall, 210 (90.1%) participants demonstrated good knowledge regarding vaginal infections and their management. Most respondents correctly identified bacterial vaginosis as the most common cause of vaginal discharge (n=152; 65.2%), recognized the typical presentation of *Candida* infections as thick, white, curd-like discharge (n=222; 95.3%), and associated trichomoniasis with frothy, yellow-green discharge (n=213; 91.4%). Regarding treatment, (n=209; 89.7%) identified antibiotics as first-line therapy for bacterial vaginosis, 223 (95.7%) recognized antifungals for *Candida* infections, and (n=217; 93.1%) correctly selected metronidazole for trichomoniasis.

In terms of diagnostic knowledge, 147 (63.1%) reported awareness of Amsel’s criteria for diagnosing bacterial vaginosis, and 167 (71.7%) acknowledged the diagnostic role of the whiff test. However, fewer participants correctly identified diagnostic methods for fungal and parasitic infections; only 137 (58.8%) recognized wet mount microscopy for *Candida*, and 96 (41.2%) reported using a saline wet mount to detect *Trichomonas vaginalis*, with a substantial proportion expressing uncertainty.

As shown in Table 2, most sociodemographic variables were not significantly associated with knowledge levels. However, a statistically significant association was found for marital status (p = 0.004), with married participants demonstrating better knowledge compared to single or divorced individuals. Similarly, professional level was significantly associated with knowledge (p = 0.023), with specialists and consultants scoring higher than general practitioners and residents. No significant associations were observed with gender, age, income, years of experience, or nationality.

**Table 2:** Knowledge Level by Sociodemographic Characteristics * Statistically significant at p < 0.05 Age is presented in years. Income categories were defined as: low (<5,000 SAR/month), good (5,000–10,000 SAR/month), high (>10,000 SAR/month). GP = general practitioner; SD = standard deviation; PHC = primary health care

Variable		Knowledge		Chi-square (χ²)	p-value
		Poor	Good		
Gender	Male	11	107	0.004	0.474
	Female	12	103		
Age (years)	20-29	16	98	4.583	0.205
	30-39	6	87		
	40-49	1	21		
	50-59	0	4		
Marital status	Single	11	38	11.177	0.004*
	Married	11	163		
	Divorced	1	9		
Professional level	GP	6	36	9.499	0.023*
	Resident	16	99		
	Specialist	0	45		
	Consultant	1	30		
Income	Low	1	5	0.623	0.732
	Good	18	157		
	High	4	48		
Experience years	1-5	19	132	3.551	0.169
	6-10	2	37		
	More than 10	2	41		
Nationality	Saudi	23	205	0.451	0.592
	Non-Saudi	0	5		

Attitudes toward vaginal discharge management were generally positive, with 157 (67.4%) participants demonstrating a favorable outlook. A majority strongly agreed that managing vaginal discharge is a high clinical priority (n=132, 56.7%), that adherence to clinical guidelines is important (n=148, 63.5%), and that ongoing education is essential (n=144, 61.8%). Additionally, 156 (67.0%) strongly supported the need for patient education, and 117 (50.2%) endorsed increased access to resources and training.

Confidence in diagnosis was moderate, with 80 (34.3%) agreeing and 69 (29.6%) strongly agreeing that they felt confident managing cases of vaginal discharge. However, attitudes toward routine STI testing were more variable; only 52 (22.3%) strongly agreed with the practice, while 71 (30.5%) remained neutral. Responses related to cultural sensitivity and communication comfort were mixed, although 117 (50.2%) strongly agreed they were comfortable discussing reproductive health issues with patients.

As shown in Table 3, favorable attitudes were more common among female participants, those aged 30-39, married individuals, and those with higher professional levels or income. However, these differences were not statistically significant, as all p-values exceeded 0.05.

**Table 3:** Attitude Level by Sociodemographic Characteristics Age is presented in years. Income categories were defined as: low (<5,000 SAR/month), good (5,000–10,000 SAR/month), high (>10,000 SAR/month). GP = general practitioner; SD = standard deviation; PHC = primary health care

Variable	Category	Unfavorable	Favorable	Chi-square (χ²)	p-value
Gender	Male	41	77	0.316	0.287
	Female	35	80		
Age (years)	20–29	43	71	4.116	0.249
	30–39	27	66		
	40–49	6	16		
	50–59	0	4		
Marital status	Single	19	30	1.462	0.481
	Married	53	121		
	Divorced	4	6		
Professional level	GP	16	26	0.712	0.87
	Resident	36	79		
	Specialist	14	31		
	Consultant	10	21		
Income	Low	2	4	0.078	0.999
	Good	57	118		
	High	17	32		
Experience (years)	1–5	50	101	0.078	0.962
	6–10	12	27		
	>10	14	29		
Nationality	Saudi	75	153	0.016	0.474
	Non-Saudi	1	4		

Good clinical practice was observed in approximately one-third of participants (n = 77, 33%). A majority reported consistently taking detailed patient histories (51.5% always) and documenting findings (61.8% always). High adherence was also noted in prescribing antifungal treatment (70%) and providing patient education on hygiene practices (60.9%).

However, several essential diagnostic procedures were notably underutilized. A substantial proportion of participants reported rarely or never performing physical examinations, applying Amsel’s criteria, or using KOH preparation and saline wet mounts for diagnosis. While antibiotic prescribing and patient follow-up showed moderate consistency, the overall findings reveal significant variability in clinical practice, particularly in the application of diagnostic protocols.

As shown in Table 4, most sociodemographic variables, including gender, age, marital status, income, years of experience, and nationality, were not significantly associated with practice level (p > 0.05). The only variable with a statistically significant association was professional level (p = 0.003), with residents more likely to demonstrate good practice compared to general practitioners, specialists, and consultants. This may reflect the influence of recent academic training and stronger adherence to structured clinical guidelines among residents.

**Table 4:** Practice Level by Sociodemographic Characteristics * Statistically significant at p < 0.05 Age is presented in years. Income categories were defined as: low (<5,000 SAR/month), good (5,000–10,000 SAR/month), high (>10,000 SAR/month). GP = general practitioner; SD = standard deviation; PHC = primary health care

Variable		Practice		Chi-square (χ²)	p-value
		Poor	Good		
Gender	Male	38	35	1.96	0.165
	Female	73	42		
Age (years)	20-29	72	42	1.554	0.67
	30-39	65	28		
	40-49	16	6		
	50-59	3	1		
Marital status	Single	30	19	1.555	0.46
	Married	118	56		
	Divorced	8	2		
Professional level	GP	35	7	14.094	0.003*
	Resident	64	51		
	Specialist	33	12		
	Consultant	24	7		
Income	Low	3	3	1.782	0.41
	Good	115	60		
	High	38	14		
Experience years	1-5	95	56	3.39	0.184
	6-10	28	11		
	More than 10	33	10		
Nationality	Saudi	152	76	0.532	0.465
	Non-Saudi	4	1		

Correlation analysis using Spearman’s rho revealed no significant relationship between overall knowledge and either attitude (r = 0.046, p = 0.485) or practice (r = -0.043, p = 0.516). However, a significant positive correlation was found between overall attitude and practice (r = 0.275, p < 0.01), suggesting that participants with more favorable attitudes were more likely to engage in appropriate clinical practices when managing vaginal discharge.

## Discussion

This cross-sectional study assessed the knowledge, attitudes, and practices of primary health care physicians in Alahsa, Saudi Arabia, regarding the diagnosis and management of vaginal discharge. Overall, the findings revealed a strong theoretical understanding among participants, particularly in identifying common infectious etiologies such as bacterial vaginosis, vulvovaginal candidiasis, and trichomoniasis. These three pathogens are responsible for the majority of infectious vaginitis cases, in line with global evidence [5,11].

Most physicians demonstrated accurate knowledge of characteristic clinical features and first-line treatments, such as metronidazole for BV and antifungal agents for candidiasis, which reflects good assimilation of standard guidelines [12]. However, gaps were evident in diagnostic competencies, especially concerning wet mount microscopy and saline testing for Trichomonas. Only a minority of participants reported consistently using these tools, despite their low cost and utility in outpatient settings. Similar diagnostic underutilization has been reported in other primary care contexts, often attributed to limited resources, cultural constraints on physical examinations, or insufficient training [13].

A significant association was found between knowledge levels and both marital status and professional rank. Specialists and consultants outperformed residents and general practitioners, suggesting that clinical experience and case exposure may reinforce awareness of guideline-based management. However, variables such as age, gender, years of experience, and income showed no significant association with knowledge, highlighting the need for broad-based continuing medical education (CME) programs accessible to all practitioner groups [14].

The majority of respondents held favorable attitudes toward vaginal discharge management. Most acknowledged the importance of adhering to clinical guidelines, providing patient education, and seeking ongoing training, elements that are critical for high-quality, patient-centered care [15]. Nevertheless, clinical practice scores remained suboptimal, with only one-third of participants routinely implementing recommended diagnostic protocols such as Amsel’s criteria or pH testing. These discrepancies between attitude and practice are well-documented and may reflect structural barriers, lack of hands-on diagnostic tools, or low prioritization in busy clinical settings [16].

Interestingly, professional level was the only demographic variable significantly associated with practice level, with residents demonstrating higher adherence to recommended protocols than more senior physicians. This may be explained by their recent academic exposure and active supervision in training environments. More importantly, a positive correlation between attitude and practice was observed, underscoring the pivotal role of motivation, confidence, and perceived importance in translating theoretical knowledge into clinical action [17]. However, the absence of a significant correlation between knowledge and practice suggests that knowledge alone is insufficient without contextual support and a proactive mindset.

These findings are consistent with patient-side research conducted among adolescent females in Riyadh, where a large proportion reported abnormal vaginal discharge, yet only a minority had adequate knowledge or sought professional care. The study cited embarrassment and fear of examination as key barriers [18]. When coupled with provider-side diagnostic gaps, these challenges highlight systemic limitations in addressing reproductive health at the primary care level.

This study is not without limitations. The cross-sectional design prevents causal interpretation, and reliance on self-reported data may introduce recall or social desirability bias, particularly concerning sensitive clinical practices. The findings are also limited to the Alahsa region and public sector physicians, which may restrict generalizability. Future research should incorporate longitudinal or interventional designs and qualitative methodologies to uncover the nuanced barriers affecting diagnostic practices. Moreover, targeted CME programs, standardized protocols, and culturally sensitive communication strategies may improve both physician performance and patient outcomes.

## Conclusions

This study found that primary health care physicians in Alahsa demonstrated strong knowledge and positive attitudes toward the management of vaginal discharge, particularly regarding common etiologies and treatments. However, clinical practice was less consistent, with limited use of essential diagnostic tools. Professional level and marital status were significantly associated with knowledge, while professional level alone influenced practice. Although knowledge did not correlate with practice, a positive association between attitude and practice was observed, suggesting that motivation and mindset play a role in care delivery. These findings underscore the need for targeted training and resource support to close the gap between knowledge and clinical implementation.

## Appendices

Questionnaires

Tables 5-8 contain the contents of the questionnaire administered to the study population.

**Table 5:** Demographic Characteristics

Variable	Categories
Age (years)	
Gender	Male / Female
Marital status	Single / Married / Divorced / Widow
Professional level	General practitioner / Resident / Specialist / Consultant
Monthly income	Weak / Good / High
Experience	≤5 years / 6–10 years / >10 years
Center name	
Nationality	Saudi / Non-Saudi
Working hours/week	

**Table 6:** Knowledge of Vaginal Infections (Yes/No/Don’t know)

Item	Response		
	Yes	No	Don’t know
Bacterial vaginosis is the most common cause of vaginal discharge.	Yes	No	Don’t know
Candida infections often present with thick, white, and curd-like vaginal discharge.	Yes	No	Don’t know
Trichomoniasis is typically associated with a frothy, yellow-green vaginal discharge.	Yes	No	Don’t know
Amsel’s criteria is a reliable diagnostic method for bacterial vaginosis.	Yes	No	Don’t know
The “whiff test” is useful in diagnosing bacterial vaginosis.	Yes	No	Don’t know
Candida infections can be diagnosed with a wet mount microscopy.	Yes	No	Don’t know
Trichomonas vaginalis can be identified through a saline wet mount.	Yes	No	Don’t know
Antibiotics are the first-line treatment for bacterial vaginosis.	Yes	No	Don’t know
Antifungal medications are effective in treating Candida infections.	Yes	No	Don’t know
Metronidazole is the preferred treatment for trichomoniasis.	Yes	No	Don’t know

**Table 7:** Attitudes Toward Vaginal Discharge Management (1–5 Likert scale) Scale: 1 = Strongly disagree, 2 = Disagree, 3 = Neutral, 4 = Agree, 5 = Strongly agree

Item				Response					
				1	2	3		4	5
Managing vaginal discharge is a high priority in primary health care.				1	2	3		4	5
I feel confident in my ability to diagnose the cause of vaginal discharge.				1	2	3		4	5
I believe it is important to follow clinical guidelines when treating vaginal discharge.				1	2	3		4	5
Cultural sensitivities should be considered when discussing vaginal discharge with patients.				1	2	3		4	5
Continuous education on vaginal discharge management is necessary for primary health care physicians.				1	2	3		4	5
Patients with vaginal discharge should always be tested for sexually transmitted infections.				1	2	3		4	5
Addressing vaginal discharge symptoms promptly can prevent complications.				1	2	3		4	5
I am comfortable discussing vaginal health issues with my patients.				1	2	3		4	5
It is important to educate patients about the causes and prevention of vaginal discharge.				1	2	3		4	5
There is a need for more resources and training on vaginal discharge management in our health care centers.				1	2	3		4	5

**Table 8:** Practices Regarding Vaginal Discharge Management (1–5 Likert scale) Scale: 1 = Never, 2 = Rarely, 3 = Sometimes, 4 = Often, 5 = Always

Item				Response					
				1	2	3		4	5
I take a detailed patient history when assessing vaginal discharge.				1	2	3		4	5
I perform a physical examination when a patient presents with vaginal discharge.				1	2	3		4	5
I use Amsel’s criteria to diagnose bacterial vaginosis.				1	2	3		4	5
I perform a saline wet mount to check for Trichomonas vaginalis.				1	2	3		4	5
I use KOH preparation to identify Candida infections.				1	2	3		4	5
I prescribe antibiotics for bacterial vaginosis based on clinical guidelines.				1	2	3		4	5
I recommend antifungal treatment for confirmed Candida infections.				1	2	3		4	5
I educate my patients on hygiene practices to prevent vaginal infections.				1	2	3		4	5
I follow up with patients after initial treatment for vaginal discharge.				1	2	3		4	5
I document all findings and treatments related to vaginal discharge in patient records.				1	2	3		4	5
